# The effects of psychological interventions on menopausal hot flashes: A systematic review

**DOI:** 10.18502/ijrm.v20i4.10898

**Published:** 2022-05-23

**Authors:** Elahe Samami, Zohreh Shahhosseini, Forouzan Elyasi

**Affiliations:** ^1^Student Research Committee, Mazandaran University of Medical Sciences, Sari, Iran.; ^2^Sexual and Reproductive Health Research Center, Mazandaran University of Medical Sciences, Sari, Iran.; ^3^Psychiatry and Behavioral Sciences Research Center, Sexual and Reproductive Health Research Center, Addiction Institute, School of Medicine, Mazandaran University of Medical Sciences, Sari, Iran.

**Keywords:** Menopause, Climacteric, Hot flashes, Psychology.

## Abstract

**Background:**

Menopause is a normal physiological phenomenon, closely identified with a great deal of physical-psychological symptoms, including hot flashes (HFs) with a prevalence rate of 20-80%. Various pharmacological and non-pharmacological interventions have been thus far practiced to reduce this common symptom of the menopausal transition.

**Objective:**

This systematic review was conducted to evaluate the effects of psychological interventions on menopausal HFs.

**Materials and Methods:**

In this review, the databases of Google Scholar, Scopus, PubMed, Web of Science, Science Direct, the Cochrane Library, and Scientific Information Database were searched applying the Boolean searching operators as well as the keywords of `hot flashes', `menopause', `psychological intervention', and `vasomotor symptoms'. Accordingly, a total number of 20,847 articles published from January 2000 to June 2019 were retrieved. After excluding the duplicate and irrelevant ones, the risk of bias of 19 clinical or quasi-experimental clinical trials was assessed using the Cochrane collaboration tool.

**Results:**

The interventions implemented in the studies on menopausal HFs included cognitive behavioral therapy, mindfulness-based stress reduction, hypnotherapy, and relaxation techniques. All of the articles reported improvements in HFs in postmenopausal women, except for 4 studies.

**Conclusion:**

Based on the findings of this systematic review, psychological interventions, especially cognitive behavioral therapy and relaxation techniques, are potentially effective for vasomotor symptoms and HFs in healthy postmenopausal women, although the quality of published research on this topic is sometimes questionable.

## 1. Introduction

Menopause is a normal physiological phenomenon in women aged 47-55 yr (1, 2). It is often closely identified with common symptoms such as anxiety, depression, stress, mood disorders, sexual concerns, night sweats, and in particular hot flashes (HFs) (1, 3-6). HFs represents the most common complication of menopause, with a prevalence rate of 20-80% (7-9). HFs are a sudden feeling of warmth in the upper body, including the face, neck, and chest, which may spread to the legs (5). This symptom of menopausal transition often results in a high resting heart rate, fatigue, nausea, increased metabolism, and anxiety (8). Studies show that some women might experience HFs for more than 20 yr. Therefore, it is of paramount importance to focus attention on this issue in women's health (8, 10).

According to the literature, various methods can improve HFs in postmenopausal women, including hormone therapy (9, 11, 12) along with the use of non-hormonal medications (e.g., clonidine and gabapentin) (8, 13), medicinal herbs (e.g., black cohosh) (14), complementary medicine (15) and psychological interventions such as cognitive behavioral therapy (CBT) (16-22), mindfulness-based stress reduction (MBSR) (23, 24), and hypnotherapy (5, 8, 25). Studies in this line have demonstrated that hormone therapy is the principal treatment for HFs in postmenopausal women. However, its continuous use might increase the risk of breast cancer, cardiovascular diseases, stroke, venous complications, and thromboembolism (4, 5). Non-hormonal treatments might lead to potential side effects and incur considerable costs in the long term (8). Concerning these complications and risks, women tend to benefit from non-pharmacological treatments such as complementary medicine and psychological interventions (7, 26).

Studies have shown that the use of psychological interventions such as CBT, MBSR, and hypnotherapies lessen the frequency and severity of HFs, enhance sleep quality, and reduce night sweats, depression, and anxiety in postmenopausal women (4, 8, 24, 27). However, some surveys have suggested that psychological interventions might fail to lessen HFs or improve menopause symptoms even though their results have been non-significant (20, 22, 28). The lack of significant findings can be attributed to different measurement tools and small sample sizes.

As the results of these studies regarding the effects of psychological interventions on menopausal HFs are contradictory, a structured, systematic review was conceived as one way to overcome these inconsistencies since it brings together a series of related investigations and allows readers to consider the results of multiple studies on the same topic simultaneously (29, 30). A comprehensive overview of the available literature in this field indicated that previous systematic reviews had solely evaluated the effects of non-pharmacological treatments (31), medicinal plants (14), and relaxation techniques (32) on HFs and other symptoms of menopause. Accordingly, no specific assessment has been performed on the effects of psychological interventions, to the best of the authors' knowledge. With this background in mind, this systematic review aimed to investigate the effects of psychological interventions on menopausal HFs.

## 2. Materials and Methods

### Research question 

This study was a systematic review carried out based on the performed reporting items for systematic reviews and meta-analyses (PRISMA) guidelines (33, 34). In addition, this systematic review addressed the following question: are psychological interventions effective in reducing menopausal HFs?

### Search strategy 

To find all the electronic articles published from January 2000 to June 2019, the databases of Google Scholar, Scopus, PubMed, Web of Science, Science Direct, the Cochrane Library, and Scientific Information Database were searched. The list of the studies retrieved was manually searched to extract the ones with related topics. The last search was done on June 22, 2019. The researchers also used the following English keywords or their Persian equivalents regarding the Boolean searching operators: (`Menopause' OR `Premenopausal' OR `Post Menopause' OR `Climacteric') AND (`Hot Flashes' OR `Vasomotor Symptoms' OR `Menopause Symptoms') AND (`Intervention' OR `Psychological Intervention' OR `Supportive Intervention' OR `Cognitive Behavioral Therapy' OR `Mindfulness' OR `Psychotherapy' OR `Behavior Therapy' OR `Relaxation' OR `Hypnosis').

### Study selection 

At this stage, all the articles related to the research question were extracted through a systematic and advanced search. The irrelevant articles were recognized based on their titles, abstracts, and full texts upon eliminating the duplicates. Article selection was done by 2 independent researchers (F.E and E.S) and in the case of a disagreement, the third researcher made the final decision (Z. Sh). All the studies considered for this systematic review met the following inclusion criteria: clinical or quasi-experimental clinical trials published, which focused on the effects of psychological interventions on menopausal HFs (naturally or surgically induced), reported sample size, and reported results of interventions. Articles about HFs in women with cancer and chemotherapy-induced menopause, those in languages other than English and Persian, and the studies whose abstracts had been presented at conferences without their full texts were excluded from this review.

### Data extraction and quality assessment 

The data including authors' names, year, study design, location, goals, age, sample size, tools, type of interventions, duration of intervention, follow-up, outcomes, and results were extracted following the abstracts' assessment and review, and full texts of the eligible articles. Finally, the extracted data were classified and reported as a systematic review and illustrated in tables I and II.

The risk of bias of the included articles was assessed with the Cochrane collaboration tool (35, 36). The risk of bias was assessed for each study using the following 7 items: random sequence generation and allocation concealment (selection bias), blinding of participants and personnel (performance bias), blinding of outcome assessment (detection bias), incomplete outcome data (attrition bias), selective reporting (reporting bias), and anything else ideally prespecified (other bias). Each item was classified as low risk, high risk, and unclear. Finally, overall rating of risk of bias is as follows: the study has low risk of bias for all items (low risk); the study has high risk of bias in at least one items for this result (high risk); and the study has to raise some concerns in at least one items for this result, but not to be at high risk of bias for any items (unclear) (35-38).

**Table 1:** Article descriptions


** Authors, year (Ref)**	**Study design**	**Location**	**Goals**	**Age (yr)**	**Sample size**	**Tools**
** Hardy ***et al.,*** 2018 (7)**	Randomized controlled clinical trial	United States	The effects of SH-CBT on working women with HFs and NS	45-60	124	HFRS, NSRS, MRQ, SPS, WSAS, WHQ
** Lindh-Astrand ***et al*** **., 2015** (28)**	Randomized controlled clinical trial	Sweden	The effects of online AR on vasomotor symptoms in postmenopausal women	47-60	46	HFRDIS
** Larroy ***et al*** **.,** 2015 (16)**	Quasi-experimental	Spain	The effects of cognitive therapeutic techniques on menopause, depression, and anxiety in Spanish women	42-55	53	HADS, BKMI
** Norton ***et al*** **.,** 2014 (17)**	Randomized controlled clinical trial	United Kingdom	The effects of CBT on vasomotor symptoms (HF and NS)	≥ 18	140	HFRS, NSRS, GHS-SF-36, WHQ, SSC, SAS, HFBehS
** Stenfonopoulu and Hunter, 2014 (18)**	Quasi-experimental	United Kingdom	The effects of SH-CBT via phone calls on menopause symptoms	44-77	47	HFRS, HFBS, HFBehs, WHQ
** Green ***et al*** **.,** 2013 (4)**	Quasi-experimental	The effects of CBGT on menopause symptoms	40-60	8	HFRDIS, GCS, MEN-QOL, MADRS, UQOLS, PSQI, HAS
** Elkins ***et al*** **.,** 2013 (8)**	Randomized controlled clinical trial	United States	The effects of hypnotherapy on treatment of HFs in postmenopausal women	≥ 18	187	PSQI, HFRDIS, HADS, HFSD
** Elkins ***et al*** **.,** 2013 (5)**	Quasi-experimental	United States	The effects of self-hypnosis on treatment of HFs in postmenopausal women	46-62	13	HFRDIS
** Saenask ***et al*** **.,** 2013 (26)**	Randomized controlled clinical trial	Thailand	The effects of AR and MR on treatment of menopausal and premenopausal symptoms	45-60	71	MRS
** Kendrick ***et al*** **.,** 2013 (25)**	Quasi-experimental	United States	The effects of HRT on HFs in postmenopausal women	≥ 18	62	HFSD, HFRDIS
** Ayers ***et al*** **.,** 2012 (19)**	Randomized controlled clinical trial	United Kingdom	The effects of SH-CBT on reducing HFs and NS in postmenopausal women	≥ 18	140	HFRS, WHQ, HRQOL, GHS-SF-36, SSC
** García and Gómez- Calcerrada, 2011 (20)**	Clinical trial	Spain	The effects of CBT on women with mild menopausal symptoms	43-56	46	BKMI, HADS, QOLAQ
** Carmody ***et al*** **.,** 2011 (23)**	Randomized controlled clinical trial	United States	The effects of MBSR on HFs	47-69	110	HADS-A, MENQOL, WHIIRS, HF-Intensity
** Kamali and Mousavinasab, 2007 (10)**	Quasi-experimental	Iran	The effects of hormone therapy and relaxation techniques on vasomotor disorders in postmenopausal women	- 90	A self-made demographic questionnaire including 3 sections: Socio-demographic characteristics, medical history, health status and vasomotor disorders
** Zaborowska ***et al*** **.,** 2007 (27)**	Randomized controlled clinical trial	Sweden	The effects of AR on HFs in postmenopausal women	- 102	BKMI
** Carmody ***et al*** **.,** 2006 (24)**	Quasi-experimental	United States	The effects of MBSR on HFs	47-60	15	MENQOL, Daily HF log, HFRDIS, WHIIRS, SCL-90-R, PSS, Taranto mindfulness scale, Mindfulness practice daily
** Alder ***et al*** **.,** 2006 (21)**	Clinical trial	Sweden	The effects of CBT on climacteric syndrome	42-65	30	MRS, PQ, HADS, MFSQ, VAS
** Nedstrand ***et al*** **.,** 2006 (39)**	Randomized controlled clinical trial	Sweden	The effects of AR and estradiol (oral) on treatment of vasomotor symptoms in postmenopausal women	48-63	30	BKMI, VAS, SCL-90, MOOD-Scale
** Keffer and Blanchard ***et al*** **.,** 2005 (22)**	Randomized controlled clinical trial	United States	The effects of CBGT on HFs in postmenopausal women	Mean: 51	19	MSQOL, WHQ HFRS
SH-stocktickerCBT: Self-help-cognitive behavioral therapy, HFs: Hot flashes, HFRS: Hot flash problem rating scale, NSRS: Night sweat problem rating scale, MRQ: Menopause representations questionnaire, SPS: Stand ford presenteeism scale, WSAS: Work and social adjustment scale, WHQ: Women's health questionnaire, AR: Applied relaxation, HFRDIS: Hot flash related diary interference scale, HADS: Hospital anxiety and depression scale, BKMI: Blatts-Kupperman index, CBT: Cognitive behavioral therapy, HF: Hot flash, NS: Night sweats, GHS-SF-36: General health survey short form-36, SSC: Simplex scientific scale, SAS: Somatic amplification scale, HFBehS: Hot flash behavioral scale, HFBS: Hot flash beliefs scale, CBGT: Cognitive behavioral group therapy, GCS: Green climacterics scale, Men-QOL: Menopause-specific quality of life, MADRS: Montgomery-Asberg depression rating scale, UQOLS: Utian quality of life scale, PSQI: Pittsburg sleep quality inventory, HAS: Hamilton anxiety scale, HFSD: Hot flash symptoms diary, MR: Modified relaxation, MRS: Menopause rating scale, HRT: Hypnotic relaxation therapy, HRQOL: Health related quality of life, QOLAQ: Quality of life assessment questionnaire, MBSR: Mindfulness based stress reduction, HADS-A: Hospital anxiety and depression scale-anxiety, WHIIRS: Women health initiative insomnia rating scale, HF-Intensity: Hot flash intensity, SCL-90-R: Hopkins symptom checklist-90-revise, PSS: Perceived stress scale, PQ: Partnership questionnaire, MFSQ: McCoy female sexuality questionnaire (German translation), VAS: Visual analogue scale						

**Table 2:** The statistical results of the Mann-Whitney U Test, paired and independent-sample t test, Wilcoxon, and repeated measures analysis of included studies

**Authors** **(Ref)**	**Type of intervention**	**Duration of Intervention**	**Follow up**	**Outcomes**	**95% CI**	**P-value**	**Effect size**	**Results**	**Quality** A**ssessment**
**Hardy **et al**. (7)**	SH-CBT	4 wk	6 and 20 wk after intervention	HF problem rating	6 wk	0.86-2.11	< 0.01a	0.77*	The intervention improved HF problem rating within 6 and 20 wk (S) In addition, the intervention led to a drop in HF frequency within 6 and 20 wk (S)	High risk
					20 wk	0.31-1.87	0.01a	0.56*		
				HF frequency	6 wk	4.10-23.91	0.01a	0.39*		
					20 wk	1.05-21.66	0.05a	0.31*		
**Lindh-Astrand **et al**. (28)**	Web-based AR intervention	9 wk	Before and immediately after intervention	HF frequency	After the intervention	-	0.09b	-	The intervention had no impact on vasomotor symptoms and HF (NS)	High risk
**Larroy **et al**. (16)**	CBT	8 session (one session per wk) for 2 hr	Before and after intervention	HF symptoms	After the intervention	-	< 0.001a	-	The CBT improved the vasomotor symptoms and decreased HFs (S)	High risk
**Norton **et al**. (17)**	CBT and SH-CBT	8 session (one session per wk) for 2 hr	6 and 26 wk after intervention	HF problem rating	CBT	6 wk	-2.88 to -1.36	< 0.001a	-	The intervention reduced HF problem rating within 6 and 26 wk (S) SH-CBT intervention decreased HFs within 6 and 26 wk (S)	Low risk
						26 wk	-2.13 to -0.54	< 0.01a	-		
					SH-CBT	6 wk	-2.86 to -1.29	< 0.001a	-		
						26 wk	-2.02 to -0.36	< 0.01a	-		
**Stenfonopoulu and Hunter (18)**	SH-CBT	4 wk (one session per wk) for 2 hr and phone counseling (twice a wk) for 15-35 min	Before, 6 wk, and 3 months after intervention	HF problem rating	6 wk	48-78	< 0.001c	-	The intervention improved and decreased the HF problem rating in menopausal women (S)	High risk
					3 months	54-86	< 0.001c	-		
**Green **et al**. (4)**	CBGT	10 wk (one session per wk) for 2 hr	Before and immediately after intervention	HF symptoms	After intervention	-	0.01a	-	The CBGT intervention improved and moderated daily HFs (S)	Low risk
**Elkins **et al**. (8)**	Hypnotherapy	5 wk (one session per wk) for 45 min	Before, and 6 and 12 wk after intervention	HF-score	6 wk	12.22-16.86	< 0.001a	-	The hypnotherapy improved and reduced HF-score and HF frequency after intervention and in follow-ups within 6 and 12 wk (S)	Low risk
					12 wk	12.60-17.54	< 0.001a	-		
				HF frequency	6 wk	38.84-47.85	< 0.001a	-		
					12 wk	36.15-49.67	< 0.001a	-		
**Elkins **et al**. (5)**	Self-hypnosis	5 wk (one session per wk) for 45 min	Before and immediately after intervention	HF-score	Immediately	11.8-25.1	< 0.001c	-	The self-hypnosis intervention reduced HF-score and HF frequency (S)	High risk
				HF frequency	Immediately	52.3-79.53	< 0.001c	-		
**Saenask **et al**. (26)**	AR and MR	AR: 12 wk (one session per wk) for 60 min MR: 12 wk (one session per wk) for 60 min and 12 wk of MR home practice (5 sessions per wk) for 15-20 min	Before, 6 wk and 3 months after intervention	HF severity	AR group	6 wk	-	0.02d	-	The AR and MR intervention reduced HF severity in women and this downward trend was higher in the MR group compared with the AR one (S) While the intervention decreased HF frequency, the results were not significant (NS)	Low risk
						3 months	-	< 0.05d	-		
				HF frequency	MR group	6 wk	-	0.58d	-		
						3 months	-	> 0.05d	-		
**Kendrick **et al**. (25)**	HRT	5 wk (one session per wk) for 45 min	Before and after intervention	HF-score	After intervention	-	< 0.01c	-	HRT intervention directly decreased HF score and frequency (S)	Unclear
				HF frequency	After intervention	-	< 0.01c	-		
**Ayers **et al**. (19)**	CBT and SH-CBT	CBT: 4 wk (one session per wk) for 2 hr SH-CBT: Completing a booklet in 4 wk	Before and after intervention	HF problem rating	CBT	6 wk	-2.88 to -1.36	< 0.001a	-	The CBT and SH-CBT interventions improved HF problem rating within 6 and 26 wk (S)	Low risk
						26 wk	-2.13 to -0.55	< 0.01a	-		
					SH-CBT	6 wk	-2.09 to -1.29	< 0.001a	-		
						26 wk	-2.02 to -0.36	0.01a	-		
**García & Gómez-Calcerrada (20)**	CBT	8 wk (one session per wk) for 2 hr	Before and after intervention	HF symptoms	After intervention	-	> 0.05e	-	The CBT intervention did not improve HF symptoms (NS)	High risk
**Carmody **et al**. (23)**	MBSR	8 wk (one session per wk) for 2.5 hr with a session (morning or evening) at the end of the 6 wk	Before, immediately and 12, 16, and 20 wk after intervention	HF intensity	After intervention	19.8-44.63	< 0.001a	-	The MBSR reduced HF intensity immediately, 12, 16 and 20 wk after intervention (S)	High risk
					12 and 16 wk	0.14-24.48	0.05a	-		
					20 wk	31.81-57.31	< 0.001a	-		
**Kamali and Mousavinasab (10)**	Relaxation techniques	5 daily sessions (every other day) of relaxation and daily practice of relaxation at home for one month	Before, immediately after and one month after intervention	HF frequency	6 wk	-	< 0.001c	-	The CBT and SH-CBT improved HF problem rating within 6 and 26 wk after intervention (S)	Low risk
					One month	-	< 0.001c	-		
**Zaborowska **et al**. (27)**	AR	12 wk (one session per wk) for 60 min	Before, one, 4, and 12 wk after intervention	HF frequency	1 wk	-0.6 to 0.1	< 0.01a	-	The AR reduced daily HF frequency within one, 4, and 12 wk after intervention (S)	Low risk
					4 wk	-1.8 to -0.1	< 0.01a	-		
					12 wk	-4.1 to -1.5	< 0.01a	-		
**Carmody **et al**. (24)**	MBSR	8 sessions (7 weekly sessions [one session per wk] for 2.5 hr and one session [morning or evening] at the end of 6 wk)	Before, 7, and 11 wk after intervention	HF severity	7 wk	-	< 0.001d	-	The MBSR reduced HF severity and frequency within 7 and 11 wk after intervention (S)	Low risk
					11 wk	-	< 0.001d	-		
				HF frequency	7 wk	-	0.05d	-		
					11 wk	-	< 0.001d	-		
**Alder **et al**. (21)**	CBT	7 wk (one session per wk) for 1.5 hr	Before and immediately after intervention	HF problem rating	After intervention	-	< 0.01e	-	The CBT reduced HF problem rating after intervention (S)	High risk
**Nedstrand **et al**. (39)**	AR	12 wk (one session per wk) for 60 min	Before, immediately after, 4, 8, and 12 wk, and 3, and 6 months after intervention	HF frequency	After intervention	4.5-7.6	< 0.001e	-	The AR decreased HF frequency immediately, 4, 8, and 12 wk, and 3 and 6 months after intervention (S)	High risk
					4 wk	3.3-6.9	< 0.001e	-		
					8 wk	2.5-4.9	< 0.001e	-		
					12 wk	2.1-3.9	< 0.001e	-		
					3 months	1.0-4.7	< 0.001e	-		
					6 months	0.7-2.5	< 0.001e	-		
**Keffer and Blanchard **et al**. (22)**	CBGT	8 wk (one session per wk) for 90 min	Before and immediately after the intervention	HF problem rating	After intervention	-	< 0.21e	-	The CBGT decreased HF problem rating and frequency, but the results were not significant (NS)	Low risk
				HF frequency	After intervention	-	< 0.21e	-		
a*t* test, bMann-Whitney U Test, cPaired sample *t *test, dWilcoxon signed-rank test, eRepeated measures, *Cohen’s d, CI: Confidence interval, SH-CBT: Self-help-cognitive behavioral therapy, HF: Hot flash, HFRS: Hot flash problem rating scale, S: Significant, NS: Non-significant, AR: Applied relaxation, CBT: Cognitive behavioral therapy, HFs: Hot flashes, CBGT: Cognitive behavioral group therapy, MR: Modified relaxation, MRS: Menopause rating scale, HRT: Hypnotic relaxation therapy, MBSR: Mindfulness based stress reduction

### Ethical considerations

This is a systematic review registered in Mazandaran University of Medical Sciences (Grant number: 5824) and the Iran National Committee for Ethics in Biomedical Research, Sari, Iran (Code: IR.MAZUMS.REC.1398.1080).

## 3. Results

### Search results and descriptions of articles 

**Figure 1:**
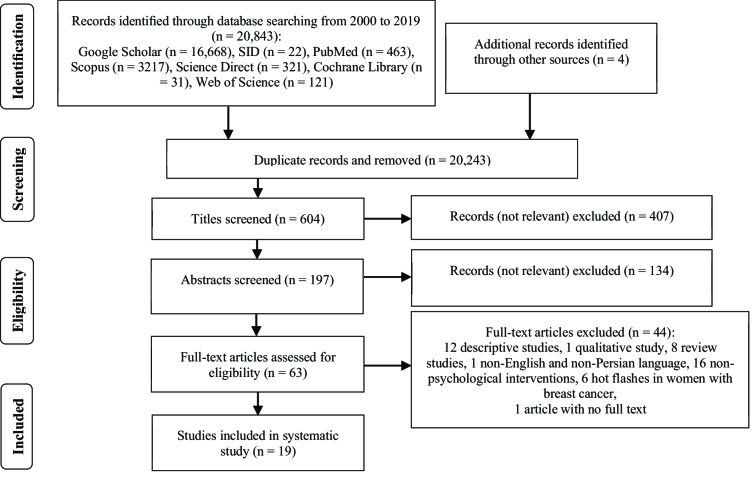
PRISMA flow diagram for selection of eligible studies.

At the first stage, 20,843 articles were obtained by searching the databases, and 4 articles were found manually. However, 197 articles remained once the duplicate and unrelated studies were eliminated. In the end, 134 and 44 articles were respectively excluded after reviewing their abstracts and reading their full texts, which led to the remaining 19 articles (Figure 1).

### Article review and study participants 

The subjects recruited in the selected articles were women who presented with the symptoms of HFs, at premenopausal, menopausal, and postmenopausal stages. Moreover, the participants' age range was 18-77 yr. (age was not been mentioned in 2 studies (10, 27)).

### Interventions 

#### CBT 

CBT is a structured, short-term, and skills-focused psychotherapy and it is a combination of cognitive and behavioral therapies. The main approach in this treatment gives priority to the role of thoughts, beliefs, maladaptive perceptions, and cognitions to attain compatible thoughts and behaviors (4, 39). 4 studies with high quality (low risk) in this systematic review evaluated the effects of CBT on menopausal HFs (4, 7, 16-22), which involved 4-10 intervention sessions held for 1.5-2 hr. The intervention sessions also provided psychoeducation about menopause and HFs, stress management, problem-solving, cognitive and behavioral strategies to help manage HFs, relaxation techniques, breathing techniques, Kegel exercises, as well as identification and control of excessive worries and irrational beliefs.

#### MBSR 

Mindfulness represents a meditation style that underlines the importance of awareness at the present moment (40). MBSR is a structured group program that uses mindfulness meditation to understand, evaluate, and deal with chronic diseases and their symptoms (24). In this review, one study with high quality (low risk) reflected on the effects of MBSR on HFs in postmenopausal women. 7 weekly sessions (one session per wk) were held for 2.5 hr, and one session (morning and evening) was organized at the end of the 6 ${}^{\text{th}}$ wk (24). During the intervention sessions, the actions included body scan meditation, intention-setting meditation, and mindfulness-stretching exercises to develop awareness (mindfulness) during movements. Besides, all of participants in the study received CDs as guided instructions for practiced mindfulness at home for 45 min, 6 days a wk.

#### Hypnotherapy

Hypnotherapy refers to one of the psychological interventions and transient states caused by changes in accuracy and focus. This notion includes changes in consciousness and memory, increased sensitivity, inclusiveness, and imaginations that would not normally be possible (8, 40). One study with high quality (low risk) in this systematic review assessed the impacts of hypnotherapy on HFs in postmenopausal women (5, 8, 25). In this study, 5 weekly intervention sessions (one session per wk) were held for 45 min. Hypnotic interventions also included induction and instruction in self-hypnosis practices towards the therapeutic goals of reduction of HFs. The participants were provided with a hypnotic suggestion for mental imagery involving coolness, safe-place imagery, hypnotic relaxation, and symptom reduction. Moreover, they were given a CD containing information about HFs for use as a home guide.

#### Relaxation techniques

Relaxation is a simple, beneficial, and executable technique in behavior therapy that can be easily performed with simple training (40). Accordingly, applied relaxation (AR) is one type of relaxation practiced in different uncomfortable situations with quick relaxation and proper breathing (28). In this review, 3 studies with high quality (low risk) evaluated the effects of relaxation techniques on menopause-induced HFs (10, 26-28, 41). In 3 articles, 12 weekly intervention sessions (one session per wk) were held for 60 min (26, 27, 41). Another study included 5 group sessions (every other day) and one month of daily relaxation practices at home (10). The intervention sessions further provided information about menopause, theories about HFs, progressive relaxation, differential relaxation, fast relaxation, release-only relaxation, application development training, and conservation programs aimed at coping with vasomotor symptoms such as HFs.

### Tools 

The outcome of HFs in postmenopausal women was measured in all of the studies. Only one study implemented a researcher-made questionnaire to assess this condition (10), and the rest utilized various standard tools. In this context, 6 studies used the Hot Flash Related Daily Interference Scale (4, 5, 8, 24, 25, 28), and 5 studies applied the Hot Flash Rating Scale (7, 17-19, 22). On the other hand, 4 studies implemented the Blatt and Kupperman Menopausal Index (16, 20, 27, 41), whereas 2 articles administered the Menopause Rating Scale (21, 26). Furthermore, 2 studies employed the Hot Flash Symptom Diary (8, 25) while one study applied the Hot Flash-Intensity scores (23).

### Article quality assessment

**Figure 2:**
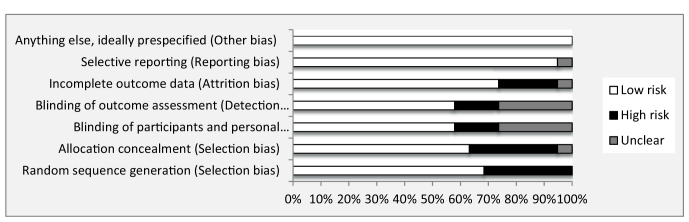
Review authors' judgment about each risk of bias item as percentages across all included studies.

**Figure 3:**
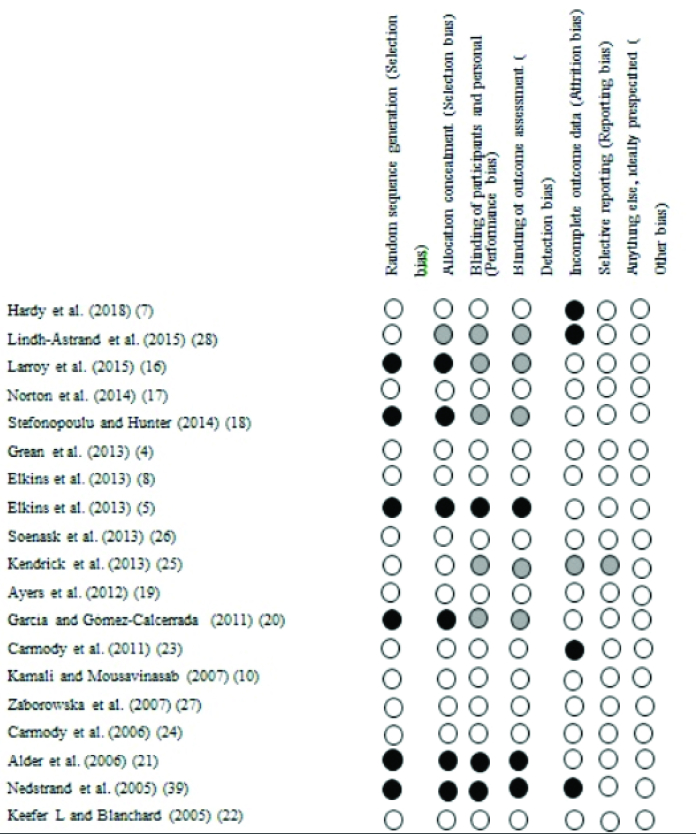
The results of the risk of bias evaluation for each included study.: 
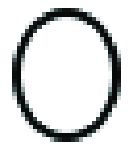
 Low risk,: 
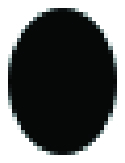
 High risk,: 
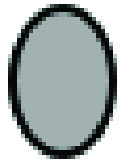
 Unclear.

The results of the quality assessment of the 19 articles conducted from 2005-2018 (18 in English and one in Persian) based on the Cochrane collaboration tool for assessing the risk of bias are presented in figures 2 and 3. The 2 reviewers (F.E, E.S) had good agreement, and any discrepancies were resolved following discussion. 6 studies had a high risk of bias in random sequence generation and allocation concealment (selection bias) (5, 16, 18, 20, 21, 39). The category of blinding participants and personnel (performance bias) was rated as at a high risk of bias in 3 studies (5, 21, 39) and low risk of bias in eleven studies (4, 7, 8, 10, 17, 19, 22- 24, 26, 27). Eleven studies had a low risk of bias in the blinding outcome assessment (detection bias) (4, 7, 8, 10, 17, 19, 22- 24, 26, 27). The category of incomplete outcome data (attrition bias) was rated as at a high risk of bias in 4 studies (7, 23, 28, 39). Only one study was unclear in incomplete outcome data (attrition bias) and selective reporting (reporting bias) (25). Finally, from the 19 articles, 9 studies were assessed as low risk (4, 8, 10, 17, 19, 22, 24, 26, 27), 9 studies had high risk (5, 7, 16, 18, 20, 21, 23, 28, 39), and one study had an unclear risk of bias (25).

## 4. Discussion

This systematic review was performed on 19 studies to evaluate the effects of psychological interventions on menopausal HFs. This was a review of studies related to various psychological interventions conducted with menopausal women. From the 19 studies, 9 studies were of a high quality and low risk of bias.

In this review and according to previous studies (7, 18, 19), CBT improved and decreased the frequency of HFs in postmenopausal women by altering thoughts and behaviors, and through a particular focus on vasomotor symptoms and HFs (4, 17).

In the present review, MBSR was also recognized as an efficient intervention targeting HFs in postmenopausal women. According to this study, MBSR decreased the frequency of daily HFs by focusing on body scan meditation and mindfulness-stretching exercises to develop awareness (24).

In this review, hypnotherapy was documented as another useful method to mitigate menopausal HFs. Hypnotherapy reduced the symptoms of vasomotor disorders and HFs through making changes in physiological responses to mental distress (25).

Relaxation was also one of the cognitive interventions affecting HFs in this review. The results of the studies showed that AR led to subsided HFs in postmenopausal women. As mentioned earlier, the findings of the studies demonstrated that relaxation, especially AR (as one the most common techniques), could moderate the frequency of HFs in postmenopausal women by moderating the sympathetic nervous system (10, 27).

The results of the included studies were fairly consistent and there was a statistically significant improvement in HF symptoms in almost all of them (4, 8, 10, 17, 19, 24, 26, 27). However, some studies were underpowered considering their small sample sizes (4, 5, 8, 22). In addition, the studies had a high risk of bias because the effects of psychological interventions are relatively influenced by the patient-therapist relationship.

The assessment of outcomes performed in some of the studies involved the comparison of a baseline assessment with a post-intervention assessment; changes found in these studies may have been due to the placebo effect (8, 27). Loprinzi et al found that placebo administration for 4 wk alleviated HFs by approximately 30% (42). Another question was related to the control group: was it enough to provide them with the usual care, or should the control group have had equal contact time as the experimental group? The answer to this question is unclear. Another potential confounding factor in this review was the time effect, because HFs decrease in frequency and intensity over time (43). To reduce the effect of this potential confounding factor, instead of using a control group, the researcher could measure the outcome at 2 different pre-intervention time points to obtain 2 baseline assessments (44).

One of the main and important problems with evaluating the effectiveness of psychological interventions for HFs is the methodological quality of the published articles, which was of impartial or poor quality and therefore precluded corrects conclusions. In order to increase internal validity, it is useful to report accurate and precise information in the study (such as the number and causes of loss to follow-up and treatment equivalence beside the trial intervention) based on the Consolidated Standards of Reporting Trial recommendations (45). Differences in outcome assessment tools used in the different studies also made it difficult to compare the results. The use of valid outcome assessment tools in all studies is crucial to ensure good external validity. Blinding of outcome assessments is another essential design feature that was often not achieved or not reported in the articles. Therefore, effective analysis was not possible due to some degree of potential bias.

According to the studies' results and the consistency of the results in the follow-ups, the CBT (4, 17, 19) and relaxation techniques (10, 26, 27) were recommended as effective low-risk treatments to reduce and improve menopausal HFs and their related symptoms. Although the results of the studies showed that MBSR (24) and hypnosis (8) therapies improved and decreased HFs in postmenopausal women as well, only one study assessed their effectiveness, which shows the need for more interventional studies on the impact of these interventions on HFs in postmenopausal women. It is necessary to mention, psychological therapies have been shown to be effective in treating other psychiatric disorders such as fibromyalgia and tocophobia (46, 47).

### Suggestions 

There is limited evidence for the impact of effective psychological therapies on symptoms of post-menopausal women. Previous studies have however shown that HF symptoms in breast cancer survivors are more common, severe and distressing than in women who have not had cancer (48, 49). Therefore, one of our suggestions is to compile a review article evaluating the effects of psychological interventions on HFs in breast cancer survivors. Further assessment on the effects of these interventions on HFs and other symptoms of menopause with high quality studies, such as randomized controlled trials, is another suggestion of this study. In addition, there is insufficient evidence to guide clinical practice for physicians, and conducting high-quality studies can be a step towards developing these guidelines.

### Clinical implications

These interventions, performed by trained health professionals, provide additional treatment options for menopausal symptoms. CBT and relaxation techniques are a low-risk treatment for HFs.

### Strengths and limitations

The major strength of this study was that it was a systematic review performed based on the reporting items for systematic reviews and meta-analyses (PRISMA) guidelines. Another strength was the use of a comprehensive search strategy in electronic databases. One of the main limitations of this study was the low number of high-quality studies available for this review. In addition, many of these studies had a high risk of bias due to the absence of adequate blinding. This bias is often an integral part of psychological studies, although evaluators may be blinded for treatment. Also, conducting a meta-analysis was impossible due to differences in and heterogeneity of the studies.

## 5. Conclusion

Based on the findings of this systematic review, psychological interventions, especially CBT and relaxation techniques are potentially effective for reducing vasomotor symptoms and HFs in healthy postmenopausal women, although the quality of published research on this topic is sometimes questionable.

##  Conflicts of Interest

The authors declare that there is no conflict of interest.
